# Carriage of colistin-resistant Gram-negative bacteria in children from communities in Cape Town (Tuberculosis child multidrug-resistant preventive therapy trial sub-study)

**DOI:** 10.4102/sajid.v36i1.241

**Published:** 2021-02-23

**Authors:** Yolandi Snyman, Andrew C. Whitelaw, Motlatji R.B. Maloba, Anneke C. Hesseling, Mae Newton-Foot

**Affiliations:** 1 Department of Pathology, Faculty of Medicine and Health Sciences, Stellenbosch University, Cape Town, South Africa; 2 National Health Laboratory Service, Tygerberg Hospital, Cape Town, South Africa; 3 Department of Medical Microbiology, Faculty of Health Science, University of the Free State, Bloemfontein, South Africa; 4 National Health Laboratory Service, Universitas Hospital, Bloemfontein, South Africa; 5 Department of Paediatrics and Child Health, Faculty of Medicine and Health Sciences, Stellenbosch University, Cape Town, South Africa

**Keywords:** colistin resistance, Enterobacterales, children, healthy, communities, Cape Town, South Africa

## Abstract

Colistin is a last-resort antibiotic against multidrug-resistant, Gram-negative bacteria. Colistin resistance has been described in the clinical settings in South Africa. However, information on carriage of these bacteria in communities is limited. This study investigated gastrointestinal carriage of colistin-resistant *Escherichia coli* and *Klebsiella* spp. and *mcr* genes in children from communities in Cape Town. Colistin-resistant *E. coli* was isolated from two participants (4%, 2/50), and *mcr-1-mcr-9* genes were not detected. Gastrointestinal carriage of colistin-resistant Enterobacterales was rare; however, continuous extensive surveillance is necessary to determine the extent of carriage and its contribution to resistance observed in clinical settings.

## Introduction

Antibiotic resistance is threatening public health globally, and colistin remains one of the last-resort antibiotics for treating infections because of carbapenem-resistant Enterobacterales. Colistin resistance is increasingly being reported in Enterobacterales both worldwide and in South Africa, which is of great concern.^1,2,3,4,5,6,7,8,9^

The predominant mechanism of colistin resistance in Enterobacterales involves changes to the phosphate groups of lipid A by adding phosphoethanolamine (PEtN) and/or 4-amino-4-deoxy-L-arabinose (L-Ara4N), resulting in reduced anionic charge of the lipopolysaccharide (LPS). Mutations in the two-component regulatory systems, PhoPQ and PmrAB, and inactivation of the *mgrB* gene are the most common causes of colistin resistance. The mobile colistin resistance (*mcr-1*) gene was first reported in 2016 in China.^1^ The *mcr* gene encodes a PEtN transferase enzyme, which transfers a PEtN to lipid A, thereby conferring resistance to colistin. Additional plasmid-mediated *mcr* genes, *mcr-2-mcr-10* and different variants thereof, have since been detected, which also confer colistin resistance.^1,2^ *mcr* genes have been mostly isolated from Enterobacterales, especially *Escherichia coli* and *Klebsiella pneumoniae,* which are responsible for both nosocomial and community-acquired (CA) infections, including sepsis, pneumonia, urinary tract infections and intra-abdominal infections^10,11^; these organisms are also common gut commensal organisms. Numerous studies have detected the *mcr* gene in isolates from clinical, community and environmental settings.^1,2,3,4,5,6^

The *mcr-1* gene has been identified in clinical isolates from at least 11 hospitals across South Africa,^7,8,9^ as well as in *E. coli* isolates from outpatients in Gauteng (*n* = 4) and the Western Cape (*n* = 1).^8^ The *mcr-1* gene has also been detected in *E. coli* isolated from broiler chickens, a pig and final effluents from wastewater treatment plants in South Africa.^3,4,5,6^ However, there are limited data regarding the carriage of colistin-resistant organisms and *mcr* genes in healthy individuals from the community. The presence of *mcr* genes in the community could serve as a reservoir for colistin resistance in clinical settings, leading to difficult and more expensive treatment for human bacterial infections, increased hospitalisation, extended hospital stays and sometimes death. Gastrointestinal carriage of plasmid-mediated colistin resistance poses additional threat as it may be transferred to other opportunistic pathogens in the gut. This study aimed to describe the gastrointestinal carriage of colistin-resistant organisms and *mcr* genes in children from Cape Town communities.

## Methodology

### Study population

Participants were enrolled in an ongoing Tuberculosis Child Multidrug-resistant Preventive Therapy (TB CHAMP) trial, a two-arm, cluster-randomised, double-blinded placebo-controlled phase-3 trial evaluating the efficacy and safety of 6 months of levofloxacin preventive therapy for treating multi-drug-resistant tuberculosis (MDR-TB), http://www.isrctn.com/ISRCTN92634082, in healthy children <5 years of age with household exposure to MDR-TB. The first 50 stool samples, collected between November 2017 and August 2018, were used. Children were enrolled from clinics based in Khayelitsha and Philippi in urban Cape Town, South Africa.

### Sample collection

Stool specimens were collected by nurses from children who were enrolled in the trial before commencing study treatment using a standard operating procedure. Six scoops of formed stool or three scoops of loose stool were collected using a 2.5 millilitres (mL) spoon (Lasec, South Africa) and mixed several times. Samples were delivered in a cooler box with an ice pack (2°C – 8°C) to the National Health Laboratory Services (NHLS) microbiology laboratory at Tygerberg Hospital and were stored at –80°C.

### Culture-based screening for colistin resistance

Stool samples were cultured on a MacConkey agar (Sigma-Aldrich, South Africa) containing 10 milligrams per litre (mg/L) vancomycin (Sigma-Aldrich) and 2 mg/L colistin (Sigma-Aldrich) to isolate colistin-resistant *E. coli* and *K. pneumoniae* using the spread plate method. Distinct bacterial colonies were selected based on their morphological resemblance to *E. coli* or *Klebsiella* spp. on the MacConkey agar. Isolates were sub-cultured on a chromogenic UriSelect agar (NHLS Media Laboratory, Green Point, South Africa) for preliminary identification, and matrix-assisted laser desorption/ionisation time-of-flight mass spectrometry (MALDI-TOF; Bruker Daltonics, Bremen, Germany) was performed to confirm species identification.

Colistin resistance was confirmed by broth microdilution (BMD) following the European Committee on Antimicrobial Susceptibility Testing (EUCAST) guidelines and clinical breakpoints version 10 (minimum inhibitory concentration [MIC] ≤ 2 mg/L: susceptible, MIC > 2 mg/L: resistant). *Escherichia coli* American Type Culture Collection (ATCC) 25922 and *E. coli* National Collection of Type Cultures (NCTC) 13846 (*mcr-1* positive) were used as colistin-susceptible and -resistant control strains, respectively.

### Detection of mobile colistin resistance genes

Genomic deoxyribonucleic acid (DNA) was extracted directly from 200 mg of each stool sample using the PSP^®^ Spin Stool DNA kit (Stratec Molecular, Germany) according to the manufacturer’s instructions. Genomic DNA was extracted from colistin-resistant colonies using a crude boil-freeze extraction method.^12^ *mcr-1 – mcr-5* genes were detected by polymerase chain reaction (PCR), as previously described.^13^ Singleplex PCR detection of the *mcr-6, mcr-7, mcr-8* and *mcr-9* genes was also performed using previously described primers,^14^ newly designed primers (MCR-9YF 5′-ATG CCT GTA CTT TTC AGG GTG AAA G-3′ and MCR-9YR 5′-TTC CGC GAA TGC CGT GGC TAA-3′) and previously described protocols.^7^ No positive controls were available for *mcr-6–mcr-9* genes.

### Ethical consideration

Ethical approval was obtained from the Health Research Ethics Committee (HREC), Stellenbosch University for the main trial (M16/02/009) and this sub-study (S17/11/269).

## Results

Bacterial growth was observed on the MacConkey agar for all 50 stool samples. A total of 70 isolates, 55 *E. coli* from 25 participants and 15 *K. pneumoniae* from six participants, were obtained on the MacConkey agar with colistin and vancomycin. *Escherichia coli* and/or *K. pneumoniae* were obtained from the colistin containing media of 30 participants. More than one *E. coli* or *Klebsiella* spp. colony was isolated from some of the participants because of differences in colony morphology, and both *E. coli* and *K. pneumoniae* were isolated from one participant. For 20 of the participants, no *E. coli* or *Klebsiella* spp. were obtained from the colistin containing media. Colistin resistance was confirmed by BMD in only 3% (2/70) of the isolates, both of which were *E. coli* (MIC = 4 mg/L). The colistin MICs of the remaining isolates were in the range of 0.125 mg/L – 0.5 mg/L. The two colistin-resistant *E. coli* were from two separate individuals, and therefore, the carriage rates of colistin-resistant *E. coli* and *K. pneumoniae* were 4% (2/50) and 0% (0/50), respectively.

The plasmid-mediated *mcr-1* to *mcr-9* colistin resistance genes were not detected in either the colistin-resistant *E. coli* isolates or the DNA extracted directly from the stool samples.

## Discussion

Colistin is regarded as one of the last-resort antibiotics, and therefore, any emerging resistance outside of hospital settings is of great concern. In South Africa, there is a lack of information regarding the carriage of colistin resistance and mechanisms of resistance in children and in the community, in general. In another Cape Town study, nasopharyngeal colonisation with resistant pathogens was reported in human immunodeficiency virus (HIV)-positive children, with extended-spectrum beta-lactamase production in 50% of Enterobacteriaceae^15^; this poses a risk of becoming infected by these resistant organisms. The TB CHAMP trial provided an opportunity to obtain data on gastrointestinal carriage of colistin resistance in healthy children from communities in Cape Town.

This study showed that carriage of colistin-resistant organisms was uncommon in healthy children enrolled from local communities, with 4% of participants carrying colistin-resistant *E. coli*. The majority (68/70) of the colonies obtained on the colistin-containing media were not colistin-resistant based on BMD. This could be related to the poor diffusion of colistin in agar as previously reported^16^ or could be influenced by the amount of stool cultured, or the abundance of bacteria in the stool sample. However, resistance was confirmed by BMD, which is considered the only reliable colistin susceptibility method.^17^ A previous study has selected colistin-resistant Gram-negative bacteria (GNB) with a higher (4 mg/L) colistin concentration in agar^18^; however, this approach has the potential to miss isolates with a borderline-resistant MIC (2 mg/L – 4 mg/L). Development of a cost-effective and user-friendly commercial BMD panel for colistin susceptibility testing or direct *mcr* screening could be valuable for surveillance purposes.

None of the *mcr-1–mcr-9* genes were detected in any of the isolates or stool samples, suggesting that although *mcr* genes have been detected in clinical settings in South Africa,^7,9^ these genes are not widely disseminated in these communities. The two colistin-resistant *E. coli* isolates could have chromosomal mutations in *pmrA, pmrB, phoP, phoQ, eptB* or *mgrB* genes, which lead to L-Ara4N and/or PEtN modification of lipid A in the bacterial outer membrane resulting in colistin resistance.^19,20,21,22,23^ In addition, other *mcr* genes (*mcr-10*) or potentially undescribed, plasmid-mediated colistin resistance genes could be responsible for colistin resistance.

To the best of our knowledge, this is the first study to investigate the carriage of colistin resistance in a South African community setting. Colistin resistance in communities, especially the dissemination of *mcr* genes, could lead to infections with these resistant organisms, which could further spread to hospital settings. Although the sample size was small, our data suggest that community carriage of colistin-resistant organisms in children is uncommon. This also reassures that colistin can still be used in this community as a last-resort antibiotic when necessary. Ongoing surveillance is needed to better understand and estimate the prevalence of colistin resistance in the community.

## Conclusion

We report a low carriage rate of colistin-resistant Enterobacterales and no plasmid-mediated colistin resistance genes in children sampled from the community level. Colistin is the last-resort antibiotic for the treatment of multidrug-resistant GNB, and therefore, there is a need to understand the prevalence of colistin resistance in community settings. Ongoing surveillance is required in additional community settings in order to determine the extent of carriage and its contribution to resistance observed in clinical settings.
