# Corrigendum: Carriage of colistin-resistant Gram-negative bacteria in children from communities in Cape Town (Tuberculosis child multidrug-resistant preventive therapy trial sub-study)

**DOI:** 10.4102/sajid.v37i1.409

**Published:** 2022-06-30

**Authors:** Yolandi Snyman, Andrew C. Whitelaw, Motlatji R.B. Maloba, Anneke C. Hesseling, Mae Newton-Foot

**Affiliations:** 1 Division of Medical Microbiology, Department of Pathology, Faculty of Medicine and Health Sciences, Stellenbosch University, Cape Town, South Africa; 2 National Health Laboratory Service, Tygerberg Hospital, Cape Town, South Africa; 3 Department of Medical Microbiology, Faculty of Health Science, University of the Free State, Bloemfontein, South Africa; 4 National Health Laboratory Service, Universitas Hospital, Bloemfontein, South Africa; 5 Department of Paediatrics and Child Health, Faculty of Medicine and Health Sciences, Stellenbosch University, Cape Town, South Africa

In the published article, Snyman Y, Whitelaw AC, Maloba MRB, Hesseling AC, Newton-Foot M. Carriage of colistin-resistant Gram-negative bacteria in children from communities in Cape Town (Tuberculosis child multidrug-resistant preventive therapy trial sub-study). S Afr J Infect Dis. 2021;36(1):a241. https://doi.org/10.4102/sajid.v36i1.241, there was an error in affiliation 1. Instead of ‘Department of Pathology, Faculty of Medicine and Health Sciences, Stellenbosch University, Cape Town, South Africa’, it should be ‘Division of Medical Microbiology, Department of Pathology, Faculty of Medicine and Health Sciences, Stellenbosch University, Cape Town, South Africa’.

The authors apologise for this error. The correction does not change the significance of study’s findings or overall interpretation of the study’s results or the scientific conclusions of the article in any way.

